# Cat and dog scavenging at indoor forensic scenes: strategies for documentation and detection

**DOI:** 10.1007/s12024-023-00762-8

**Published:** 2023-12-16

**Authors:** Lara Indra, Christian Schyma, Sandra Lösch

**Affiliations:** 1 Department of Physical Anthropology, Institute of Forensic Medicine, University of Bern, Murtenstrasse 26, CH-3008 Bern, Switzerland; 2 Department of Forensic Medicine and Imaging, Institute of Forensic Medicine, University of Bern, Murtenstrasse 26, CH-3008 Bern, Switzerland

**Keywords:** Scavenging, Forensic scene, Indoor, Cat, Dog

## Abstract

Vertebrate scavenging on human remains is occasionally observed at indoor forensic scenes, especially when pets have access to the body and their deceased owners were socially distanced. Pets feeding on corpses have implications for the forensic investigation, e.g. for trauma analysis and the assessment of the cause of death, the estimation of the postmortem interval (PMI), or the recovery of the complete set of remains. Documentation of potential scavenging in forensic practice is tenuous and needs to be improved in order to be able to use the information for future casework. Investigators need to be aware of the alterations pets can cause to human remains and how these affect further analyses. Following a combined literature review for cat and canine scavenging, we present seven new cases from Switzerland with cat and/or dog involvement. We then created a flowchart guide for a systematic collection of data to use at indoor forensic scenes of suspected scavenging. Our literature review revealed the challenge in discriminating between scavenging by domestic cats and dogs, based on the appearance of the lesions alone. Furthermore, the information that is often routinely collected in indoor fatalities with potential scavenging activity is not sufficient to perform this separation. To provide a practical basis for cat and canine scavenging and its differentiation, we summarise strategies and present a flowchart to use in forensic casework of suspected indoor scavenging.

## Introduction

Vertebrate scavenging on human remains is a frequent occurrence in forensic casework worldwide [1–4]. At indoor forensic scenes, corpses may be exposed to pets as well as to intruding vertebrates. Animals can modify the site by feeding on the remains or by scattering and removing body parts and associated evidence. In such cases, knowledge about scavengers and their behaviour supports the investigation with the search and recovery of human remains or trauma analysis [5, 6]. In addition, recognition of scavenging can prevent a falsification of the postmortem interval (PMI) estimation by avoiding methods that are affected by scavenging [7]. Furthermore, findings can be misinterpreted if animal scavenging on a corpse goes undetected. For example, animals may feed at places of injuries and thereby destroy potential information on the cause of death [8–12]. In addition, animals can cause lesions themselves, mimicking perimortem injuries or postmortem human-inflicted mutilation, such as decapitation, sharp-force impacts, scratch marks or gunshot pellet wounds [11, 13–15]. In some cases, it can be mandatory to distinguish between human and animal bite marks [16]. Furthermore, the impact of the vertebrates can alter the decomposition process: by removing soft tissue, decay rates are accelerated [11, 17, 18], and the taphonomic process slows down when they consume decomposer organisms on the body [9, 19]. Therefore, the usual methods to estimate the PMI, e.g. [20, 21], may no longer be applicable. Also, scavenging animals often disarticulate and remove body parts which impedes the recovery, investigation and interpretation of the remains [22, 23]. For the above reasons, it is important to be aware of postmortem animal activity that might have altered the body or scene. Further, it is necessary to identify the scavenger genus, or species, to be able to interpret the findings and reconstruct circumstances at and after death [9, 24].

The diversity and number of potential scavengers is more limited in indoor contexts than outdoors, especially if closed entrances restrict access from outside. However, certain factors seem to make corpses prone to be scavenged indoors. These include the presence of freely moving pets, conditions that can lead to sudden death, social isolation and unkempt living spaces [25–29].

With this article, we want to address three points that will lead to an improved data basis for indoor scavenging incidents in the long term. First, we conduct a brief literature review of indoor scavenging cases of cats and dogs. Until now, a review of these two animal taxa together is not available yet. However, we think it is required to summarise information on both combined in one paper, as they are the most numerous mammal pets in many countries and, as forensic practice shows, the simultaneous presence of both cats and dogs at a scene is common. Second, we present a retrospective collection of indoor forensic cases from Switzerland with suspected scavenging by pets. With these examples, we want to show the current state of documentation regarding scavenging in indoor scenes, and highlight the need for a systematic approach. Third, we provide a flowchart guide with variables to consider in future potential indoor scavenging incidents. With this chart, we aim to standardise the detection and handling of postmortem animal scavenging in practical casework and enhance the baseline data available for comparative analyses.

In the forensic literature, few indoor scavenging incidents are reported for cats [25, 30, 31] and only one for pet rodents, a hamster [26]. Most incidents are reported for canines [25, 32–34].

### Cats

Cats are the most numerous mammal pets in many countries, including the UK, Germany and the USA. Cat bites and scratches are frequently reported (e.g. [35, 36]). Despite that, only few cases of them scavenging human remains can be found in the forensic literature [37, 38]. Cats are generally described as being hesitant towards scavenging, especially compared to dogs [33, 39, 40]. The articles reporting domestic cat scavenging present similar findings, mainly that cats seem to consume skin and soft tissue only (Table 1). They do not show interest in bones, with the exception of phalanges that were probably collateral damage of soft tissue consumption [25, 30, 31, 40]. The reported domestic cats particularly aimed for regions such as the face, head and neck [25, 31], the upper limbs [25, 30, 31, 40] and the chest [30, 31, 40]. As Rossi et al. [25] and Byard [31] report, internal organs of neck and thorax, such as heart, lungs, larynx, esophagus and liver, seem to be consumed regularly. However, both cases involved a clowder of cats (at least ten individuals).

**Table 1:** A summary of scavenging characteristics by domestic cats and dogs as collected from the literature

	**Cats (*Felis catus*)**	**Canines (*Canis lupus familiaris*)**
Preferred areas	Head (esp. face) Neck Upper extremities Chest	Head (esp. face) Neck (additionally to face) Upper extremities Abdominal region Lower extremities Genital area
Preferred soft tissue	Skin Muscle Fat Internal organs	Skin Muscle Fat
Soft tissue removal	Stripped-off, layered	Considerable amounts
Scavenging of bone	No (small bones as collateral damage)	Yes
Amputation	No	Yes, mainly fingers/hands, also feet
Lesion margin	Ragged	Irregular, ragged and crenulated edges
Skin around lesion	Intact or irregular defects (e.g. punctures) in periphery	Covered with punctures and linear, scratch-like abrasions, also distributed over further body

The damages observed vary slightly between studies, depending on circumstances such as the number of cats, the presence of clothing and the postmortem interval (PMI). When cats scavenge, they can strip-off skin, remove the soft tissue layer by layer and leave the bones intact [31], see Table 1. Fingers can be left without tips and nails and small bones, such as phalanges, can be damaged and dispersed [30, 31]. While Byard [31] reports ragged skin defect margins but intact skin around this from an indoor scene, Garcia et al. [40] present irregular defects (e.g. punctures) at the periphery of the scavenged area along the defect margins in an outdoor setting.

### Canines

Dogs are the most favourite pet, e.g. in the UK, Germany, and the USA, judged by the number of households that keep a dog [37, 38, 41]. Many case reports of postmortem feeding on corpses in indoor settings have been published [8, 11, 14, 25, 27, 29, 32–34, 39, 42–45]. The size of the dogs ranges from small (e.g. chow, pug) to large (e.g. German shepherd, red setter), and as far as known, the postmortem interval varied from less than 1 h [8, 43] to about 4 weeks [34]. In most of the cases, cause of death was natural, and several articles explicitly mention that food sources other than the human body were available to the pets [8, 33, 42]. This contradicts hunger as the sole reason for feeding. Other authors state that dogs may lick the decedents in an attempt to wake them up, starting to act emphatically by lack of reaction and finally bite the body [8, 25, 46].

The body regions targeted by canines are more or less consistent. As the summary by Colard et al. [46] of indoor scavenged corpses shows, the primary target is the head, including the face and skull (Table 1). According to the same study, the second most frequently consumed region is the neck, followed by the upper extremities, the abdominal region, the lower extremities and the genital area in descending frequency, sometimes with complete amputations of hands or feet [46].

The lesions described in the literature usually involve the removal of a considerable amount of skin and subcutaneous fat tissue in certain exposed regions, e.g. the face and neck [11, 25, 39]. Neck lesions seem to be present only additionally to face scavenging [46]. The skin area around the main defect is partially curved and covered with punctures and linear scratch-like abrasions, while these scratches, most likely from the dog’s claws, are also found on other exposed skin areas [29, 33, 42, 43]. The wound margins of the large defects generally exhibit irregular, ragged and crenulated edges [27, 33, 39], see Table 1.

## Material and methods

We present a retrospective collection of casework from the Institute of Forensic Medicine in Bern, Switzerland, from 2004 to 2022, where corpses at forensic indoor scenes showed potential animal-inflicted lesions. The cases result from a keyword search in the institutes database using the keywords “Katze” (= cat), “Hund” (= dog), “Haustier” (= domestic pet) and “Tierfrass” (= animal scavenging), and a subsequent elimination of ones without signs of scavenging. For every case, we extracted the following information from written medico-legal reports and photographs: basic demographic data of the deceased (sex, age-at-death), clothing (undressed, lightly clothed, fully clothed), postmortem interval (PMI; short: 1–24 h, medium: 24 h–1 week, long: more than 1 week) after [46], cause and manner of death, the location of scavenging injuries, the appearance of the skin around lesions, pets being present and the presence of faunal evidence, such as scats or fur. In some cases, both, cats and dogs were present or had access to the remains. Due to the legal irrelevance, no systematic attempt has been made to identify the exact species responsible for the damage. In this article, we retrospectively examined the morphology and pattern of lesions in these ambiguous cases and compared them with the scientific literature (summarised in Table 1) to assign the lesions to either cat or canine. In the following, we assessed how data of these indoor scavenging cases from “everyday practice” can inform future casework, especially the distinction between perimortem and postmortem lesions, and the distinction between scavenging of cats and canines. Consequently, we are developing a flowchart to use at future indoor scenes of suspected scavenging, with which the data basis necessary for such distinctions can be improved.

## Case reports

Firstly, we describe the findings of seven cases in sections and provide images of assumed scavenged lesions. In the following, we provide a summary of the most important data in form of a table (Table 2).

**Table 2:** Summary of the seven cases. *h* hours, *d* days, *w* weeks. PMI as suggested by Colard et al. [46]: short – 1–24 h, medium – 24 h–1 w, long – more than 1 w

Case	Age	Sex	Clothing	PMI	Cause of death	Location of injuries	Skin around lesions	Pets present	Faunal evidence	Other
1	53	M	Full	Medium (2–5 d)	Gunshot	Face, ear	Punctures, scratches	2 cats 1 dog	-	Gunshot to chest
2	60	F	Light	Medium (1–4 d)	Unclear (suspected natural)	Face, neck, genital region	Intact	1 dog	-	-
3	66	F	Full	Short (4–13 h)	Natural	Face	Few parallel scratches	1 dog (pug)	-	Unkempt house
4	53	M	Unclothed	Long (2–3 w)	Gunshot	Skull incl. face, right arm and leg, right hip region	Intact	3 dogs	-	Gunshot to skull
5	69	F	Unclothed	Long (several days to weeks)	Cardiac arrest	Left hand and wrist	Intact, possibly single punctures	4 dogs 2 cats	-	-
6	51	F	Light	Medium (several days)	Internal bleeding	Genital region	Intact	2–3 cats 1 dog	Faeces, animal hair	-
7	58	F	Full	Long (several days to weeks)	Unclear (suspected natural)	Genital region	Occasional punctures and scratches	1 dog	Animal hair	Clothing of lower body pulled down

### Case 1

**Fig. 1:**
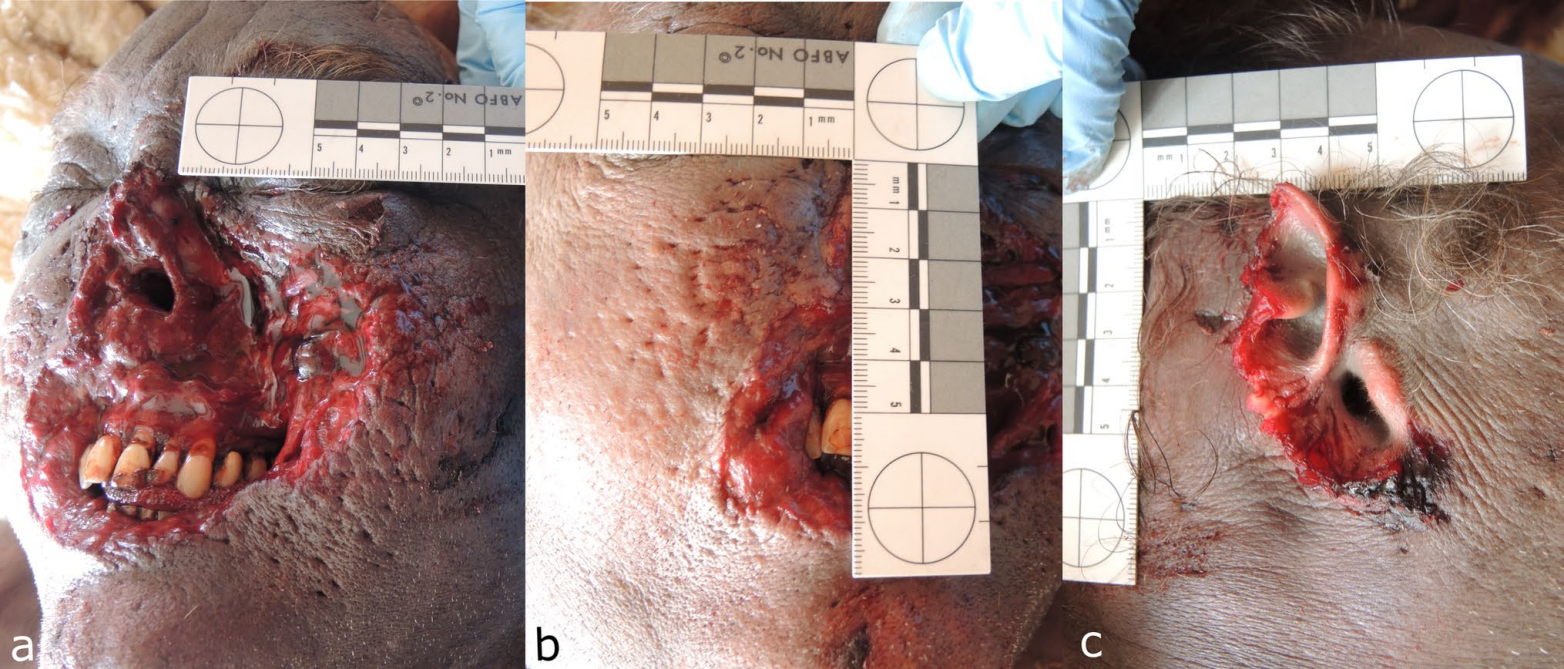
Lesions of case 1 **a** in the area of nose and mouth with **b** surrounding pits and punctures and **c** the right auricle partially removed

A corpse of a 53-year-old man was found in the bed of his house in October. He was clothed in trousers, a shirt, pants and socks, and was in the fresh decomposition stage. A medium PMI was estimated of at least 2 days due to decomposition and a maximum of 5 days due to last contact alive. The deceased had a gunshot wound to the left chest and considerable soft tissue damage to the nose, mouth and right ear, with the nasal structures, lips and about half of the right auricle missing (Fig. 1). The margins of the lesions were frayed and irregular, and the skin around the face and ear lesions had numerous “pinhead-sized” circular punctures. Cause and manner of death were determined to be a suicidal gunshot wound to the chest, and no autopsy was performed. Two cats of the deceased as well as a dog had access to the corpse. However, there was no information on the dog breed or size available.

### Case 2

**Fig. 2:**
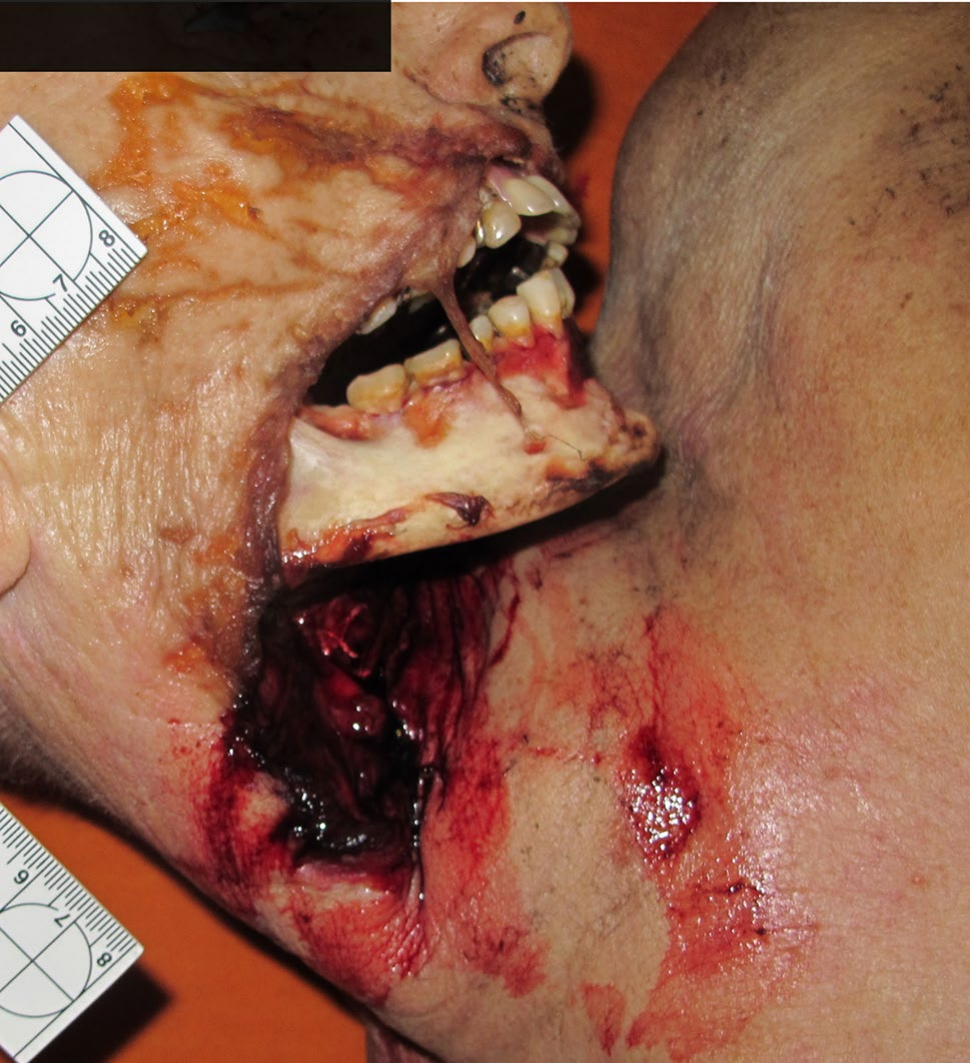
Indoor dog scavenging resulting in removal of the neck organs and exposure of the mandible in case 2

A 60-year-old woman was found dead on the bed of her one-room flat during October. She wore a long-sleeve jumper but otherwise was unclothed. Her body was in the fresh decomposition stage and a medium PMI was estimated of a minimum of 1 day due to decomposition and a maximum of 7 days, due to last contact alive. Soft tissue was removed from the lower face and upper anterior neck region, including the neck organs, leaving the mandible completely and the maxillary bone partially skeletonized and the skin around the defect with numerous, seemingly unordered scratches (Fig. 2). In the genital area, e.g. on the inner thighs and pubic mound, desiccated laminar skin lesions were found. The cause and manner of death remained unclear; however, based on the medical history of the deceased, a natural cause of death was suspected and no autopsy was performed. A small dog, resembling a miniature pincher, was in the apartment and had access to the corpse.

### Case 3

**Fig. 3:**
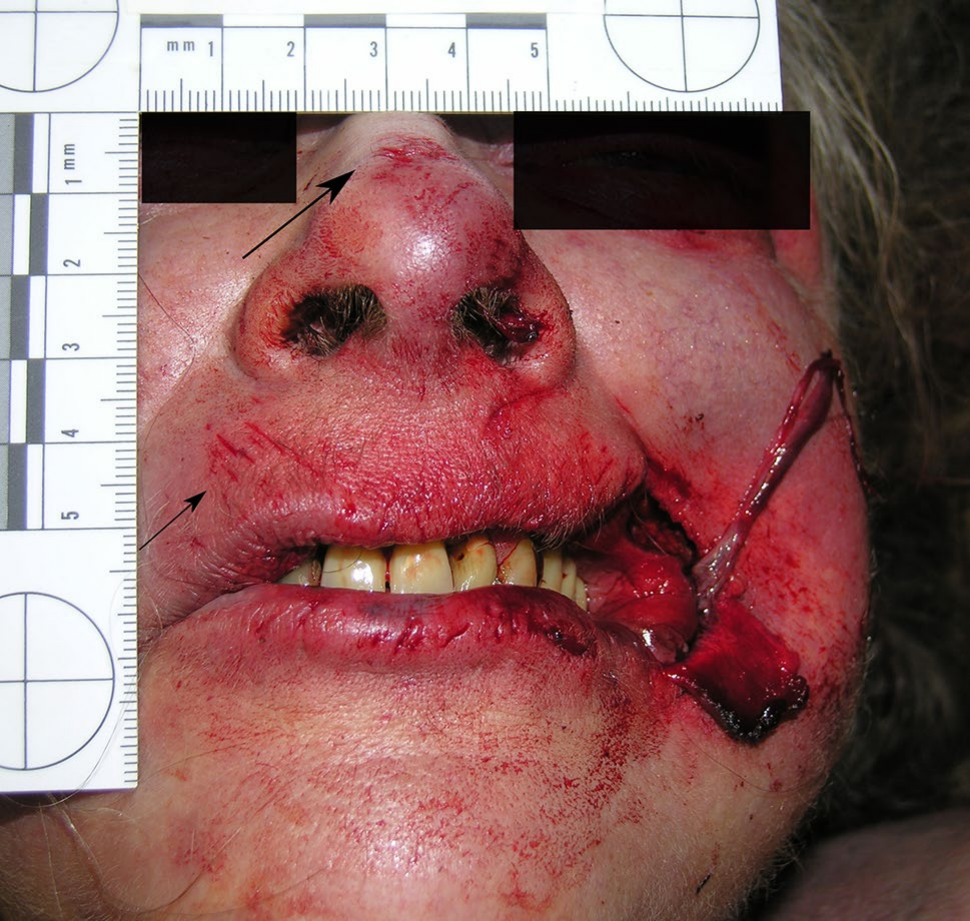
Soft tissue lesions in the right corner of the mouth in case 3. Note the scratches above the upper lip and on the nose back (arrows)

A 66-year-old woman was found dead on the floor in her flat during December. The rooms were “cluttered” and unkempt. She wore a long-sleeve shirt, trousers, stockings and socks, and her body was in the fresh decomposition stage. A short PMI was estimated of 4 to 13 h based on early decomposition signs. There were scratches on the bridge of her nose and soft tissue damage around the left corner of the mouth, exposing blood vessels and muscle tissue (Fig. 3). Five parallel short scratches (approximately 3–5 mm) can be seen above the right upper lip. The autopsy revealed a natural, heart-related cause of death. When the police arrived at the scene, a pug was found sitting next to the corpse. There were dog treats lying around in the flat.

### Case 4

**Fig. 4:**
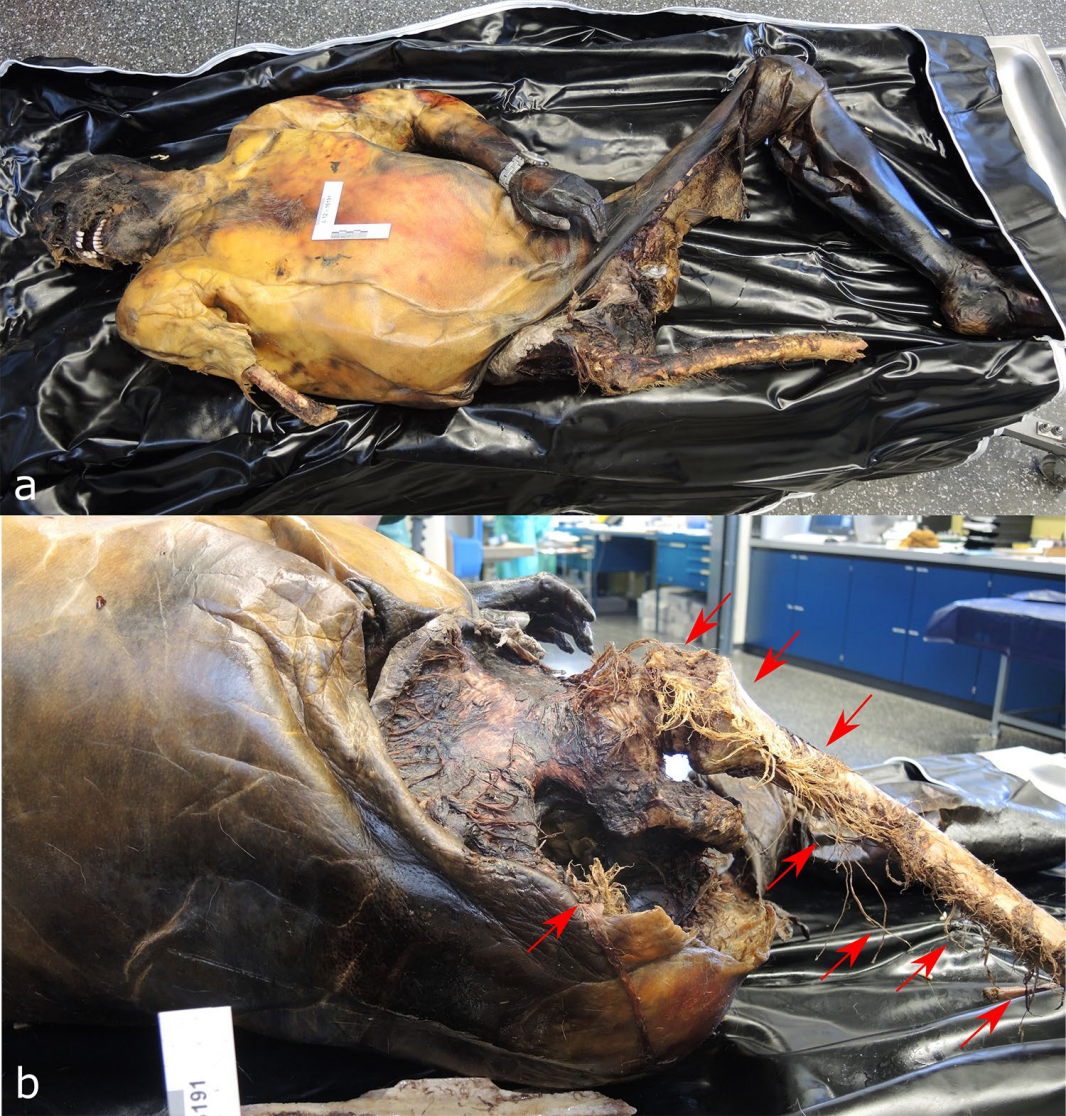
**a** The partially mummified body of a man scavenged by dogs in case 4. **b** Note the fibrous tissue remains attached to the bones (arrows)

A 53-year-old man was found dead on the floor in his flat in December. His unclothed body was partially mummified. The PMI was estimated to be long, about 2–3 weeks due to decomposition. An entry gunshot wound was present in his right forehead, an exit wound was not found and no CT imaging was performed. Soft tissue is missing from the right side of the skull, the right arm, the lower abdomen and genital region; the right pelvic area, the right leg and the bones are partially exposed (Fig. 4). The skin margins are frayed and irregular; the skin surface near the margins is intact. The arm bones are missing from the distal right humerus downwards and the leg bones from the distal right femur downwards, an approx. 20-cm-long shaft fragment of the right fibula was found nearby the body. The distal femur bone end was irregularly fractured with pits near the fracture line. The distal end of the fibula shaft showed a spiral fracture. Especially in the area of the pelvis and the right upper thigh, the mummified tendon and ligament remains are preserved as frayed fibres. The cause and manner of death was a suicidal gunshot to the head; no autopsy was performed. There were three large dogs in the flat, two Belgian and a German shepherd.

### Case 5

**Fig. 5:**
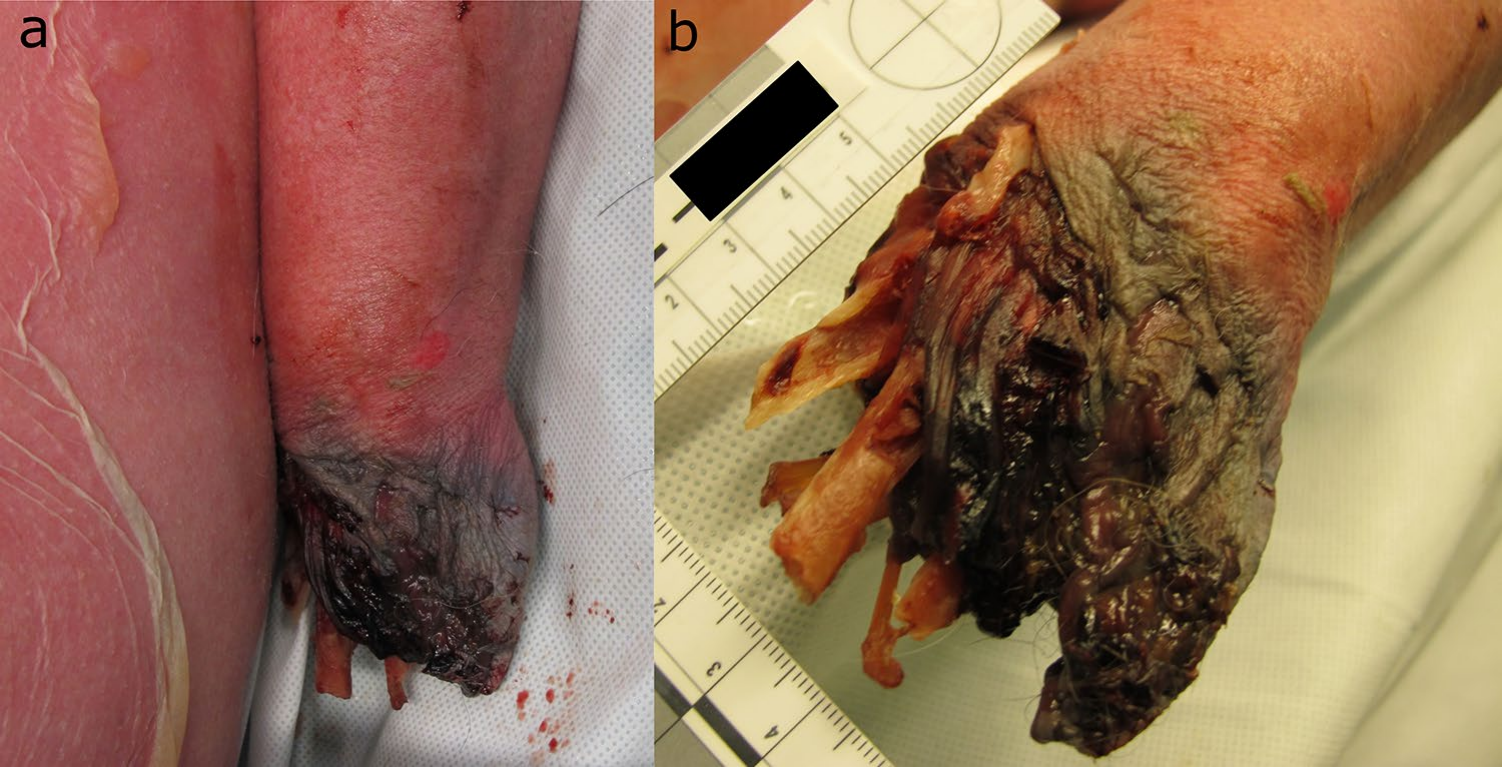
**a** The partially removed left hand of the corpse in case 5. **b** Note the bone lesions and destruction of tissue

The unclothed body of a 69-year-old woman was found during January in the bathroom of her locked house in a fresh to active decomposition stage. Based on the observed decomposition signs, the PMI was estimated to be long, from several days to weeks. Her left hand was partially missing, including the distal parts of the metacarpal bones, which were splintered (Fig. 5). The wound margin of the soft tissue was irregular and frayed, although the skin near the margin remained intact. Her death was classified as natural; no autopsy was performed. Four dogs and two cats were living in the house and had access to the corpse. However, there was no information on the dog breed or size available.

### Case 6

**Fig. 6:**
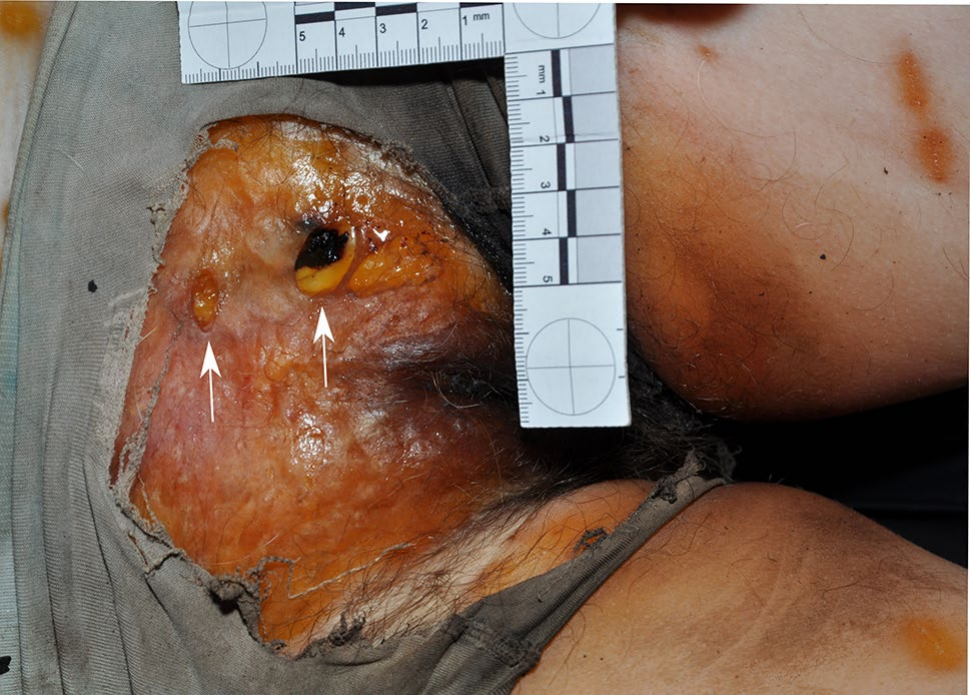
Two sharply defined oval lesions (arrows) underneath the ripped pants in case 6

The body of a 51-year-old woman was found on a couch in her locked house in January. The corpse was clothed in a t-shirt and pants and was in the fresh to active decomposition stage. Due to the advanced decomposition and the last contact alive, a medium PMI was estimated of “several days”. The pants were ripped and revealed two sharply defined oval lesions (ca. 2 × 0.5 cm and 1 × 0.5 cm) on the soft tissue, exposing the underlying fatty tissue (Fig. 6). On the inner thigh and lower abdomen, there were ca. 2-cm broad and several centimetre long scratch-like abrasions. The autopsy results indicate a natural death due to gastrointestinal bleeding. A dog and “two or three” cats were found in the house, which had access to the body. In addition, there were lots of short white hair on her t-shirt. Cat and dog faeces were present in numerous places of the premises. However, there was no information on the dog breed or size available.

### Case 7

**Fig. 7:**
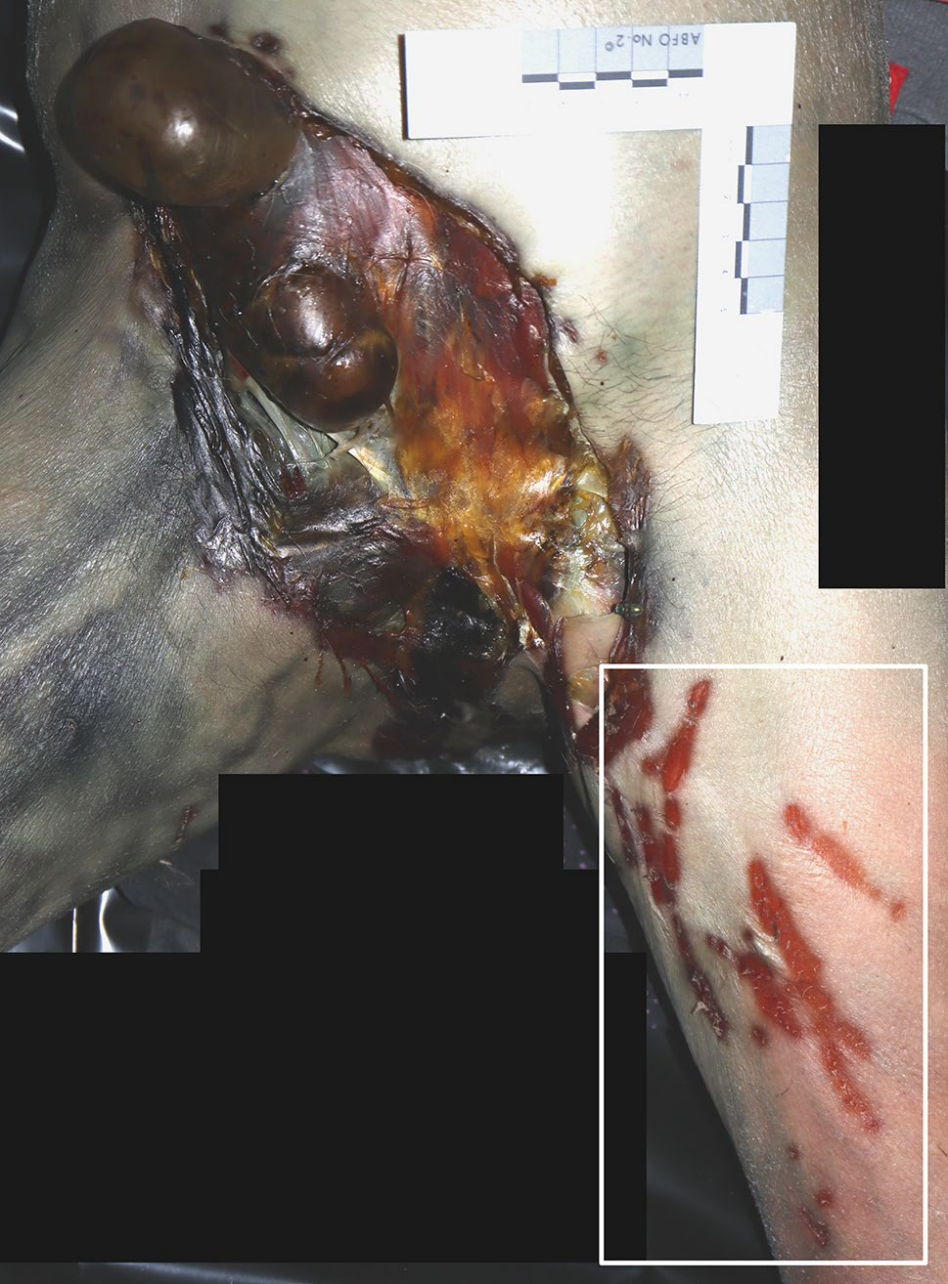
The genital region with skin defects, exposing internal structures, and the superficial skin scratches (white square) in case 7

The body of a 58-year-old woman was found on the floor in her locked apartment in October. Her corpse was clothed in a t-shirt, pullover, underpants and pants, with the pants pulled down to the ankles and the underpants half down the upper thighs. Due to the beginning active decomposition stage and the last entry in a diary, the PMI was estimated long, from several days to a few weeks with a probable maximum of 1 month. The skin of the lower abdomen, the genitals and the anus were missing with exposed fatty tissue and internal organs. The lesion margins were smooth and in two areas, grouped skin punctures of ca. 0.5–1 cm size were next to it. On the left inner thigh skin, there were several superficial, mainly non-continuous striations of ca. 0.5–1 cm breadth and 4–7 cm length, positioned in various directions. The cause of death remained unclear, but no autopsy was performed because of a history of drug abuse and a diagnosed heart disease. Therefore, a natural death was suspected. A border collie-rottweiler mix with access to the body was present in the apartment. Numerous hairs were found around the corpse and on the inside and outside of the clothing, as well as several spots with urine and faeces dispersed within the apartment, all presumably of faunal origin (Fig. 7).

In our retrospective case collection, none of the methods mentioned above could be performed to determine the actual scavenger species. In the following, we re-evaluated the reports and photographs of cases 1, 5 and 6, attempting to assign the lesions to either cats or canines.

In case 1, nose, mouth and one ear were scavenged which are typical scavenging locations for both, cats and dogs. The pinhead-sized, circular punctures concentrated near the lesion margins indicate relatively small and pointed teeth, as e.g. found in cats. In addition, the lack of widely distributed defects such as punctures and scratches in the remaining face or body parts is typical for cat scavenging [25, 40]. The case report of canine scavenging by Verzeletti et al. [39] resembles our case but presents with sometimes larger and less circular punctures near the main lesion, a more widespread distribution of these lacerations in the entire face and no scavenged ear. However, the authors do not refer to the size or breed of the dog and this information is also missing in our reports. While the overall wound morphology and pattern in our case suggest cat(s) as scavenger, verification of this hypothesis would have required further analysis, such as species-specific antigen or DNA testing of the wounds.

The partial amputation of the left hand in case 5 comes with inordinate destruction of soft tissue and bones. While cats do scavenge upper limbs, the bones remain intact and the focus is on other parts of the arm than the hands [13, 25, 30, 31, 40]. For canine activity however, the partial or complete amputation of hands including the splintering of bones is a typical finding [46]. The location and destructive nature of the lesion strongly point towards dog as the scavenging agent. A feasible method to support this hypothesis would be to analyse the faeces for bone remains.

Case 6 showed defects in the genital region, an area prone to indoor canine depredation but so far not reported for cat scavenging activity [33, 46, 47]. While the observed lesions are neither indicative for any of the species, linear scratch-like abrasions, as present on the man’s inner thigh, are a typical finding with canine scavenging [29, 43]. Further analyses such as antigen testing and DNA analysis on the lesions would have been necessary to assign the scavenger.

## Discussion

We have collated our cases retrospectively for this study. Therefore, they only contain the information that was considered important for casework from the perspective of the time or the personal processors. In the course of our study, we discovered the deficits of these processes: Crucial information such as the dog breed and size, exact number of pets at the scene, presence of faunal evidence, detailed analysis of the lesions and affected anatomical structures is often missing. However, these data would be particularly important for comparative analyses in cases where scavenging is only suspected and where a differentiation must be made between inflicted perimortem wounds and postmortem lesions. The same applies to the differentiation of scavenging by cats or dogs. Scavenging lesions are frequently handled as findings with the necessity to mention and describe but not systematically investigate. As a result, even the specialised literature rarely contains all the information required for investigations in ambiguous cases. While scavenging is often not of prior investigative importance, it can be crucial in some cases and for these, a proper data basis is important. With our literature review and cases, we therefore uncover frequently unrecorded variables in indoor scavenging cases and provide a graphical tool to overcome them.

Based on the information available from our internal case reports and available information in scientific literature, we were not able to distinct between cat and dog scavenging in the presented cases 1, 5 and 6. In this context, we also found out that systematic studies that examine differences in scavenging patterns between small and large canines, or between small dogs and domestic cats are missing. That would be relevant, since one species might mask lesions caused by the other species, and multiple involved animals further complicate the analysis. Furthermore, it is crucial to note the number, size and breed of dogs present even in cases where this information is not considered important, in order to build a solid data basis for future casework.

Some approaches can aid identification of the particular scavenger species or individual (see Table 3). We excluded the conventional bite mark analyses, because they usually aim to evaluate whether the mark is of human or animal origin, or if a specific dog inflicted the mark [16]. However, the lesion pattern resulting from (perimortem) attacks differs from (postmortem) scavenging because of the circumstances. From attacks derive relatively short-termed lesions, mostly with the intension of defence [16], whereas scavenging lesions derive from long-termed events over prolonged periods. In addition, scavenging causes more often overlapping marks and more likely greater damage than single bite incidents. The question of whether a cat or a canine is responsible therefore appears to be more difficult to answer.

**Table 3:** Methods for identifying the scavenger species responsible for lesions on a corpse when more than one species is present

Method	Reference
Examination of lesion and injury pattern	Literature for relevant species
Examination of animal faeces and vomit	[34, 39, 42, 43]
Human blood around animals mouth/paws	[33, 42]
Presence of hair/fur in or around wound	[8]
Species-specific antigen testing at lesion	[39]
Scanned 3D-model of animal’s teeth	[44]
DNA analysis	[51]

According to the literature, “risk factors” for indoor scavenging include free-roaming animals, sudden death, social isolation and disorders associated with unkempt living spaces [25–29]. Our study supports this and further suggests age over 50 as another indicator, as seen in all our examples and an above-average proportion in the literature cases.

Our cases further comply with the findings in the review by Colard et al. [46] in several aspects. For instance, most deceased have a PMI of over 24 h. Based on our cases, we suggest that scavenging presence and severity increase with a longer PMI. As in the review by Colard and colleagues, the presence or absence of clothing does not seem to be a contributing factor. In our cases, arm and hand scavenging is only present in unclothed bodies, but otherwise we cannot see a connection between presence, kind and location of clothing with scavenging intensity. The location of our defects also follows the findings reported by Colard et al. [46], with face and skull being the area most often affected. However, as opposed to their review, our decedents are female in five out of seven cases, opting for a sex bias. Nevertheless, our sample size is too small to argue for a general trend.

The remains in our case 4 were largely mummified and due to scavenging, partly skeletonized. In some areas, particularly at the skull and the exposed right femur, the remaining soft and connective tissue is fibrous and frayed, leaving single strands of several centimetres length. This appearance has been attributed to bird scavenging before, e.g. by corvids and gulls due to their pecking and tearing feeding style [10, 48, 49]. However, large dog breeds caused the lesions here, namely Belgian and German Shepard. To our knowledge, no similar findings have been reported so far from canine scavenging, although some of the human remains were also mummified [28, 50].

**Fig. 8:**
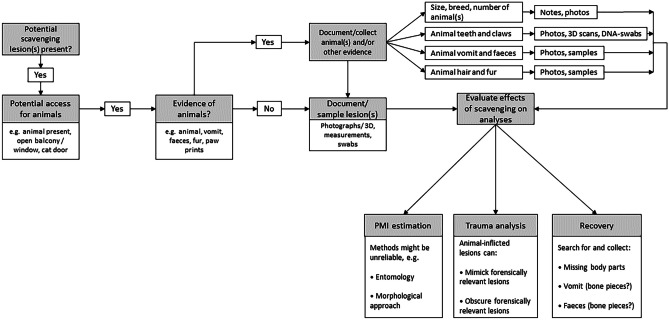
Flowchart for use at indoor forensic scenes containing human corpses with potential scavenging lesions. It shall help to identify and document evidence of scavenging, its possible influence on the analyses and further procedures at the scene

Due to the lack of documentation from our previous casework and the lack of comparable data in the literature, we could hardly draw any concrete conclusions about the scavenger(s) or differentiate between them. We therefore recognise the need to take a systematic approach for data collection in such cases. To this end, we have developed a decision diagram for indoor casework where scavenging by vertebrates is suspected (Fig. 8). This flowchart, based on scientific literature and our own observations, can aid forensic practitioners in indoor death scenes to detect and classify scavenging, and collect the necessary data for further analyses. Our diagram helps to decide, firstly, whether scavenging is a possible factor, secondly, to what extent it might influence further investigations (e.g. trauma analysis) and thirdly, what additional courses of actions are required (e.g. documentation of the animals present, DNA analysis of the lesions). To this end, we have extracted information that was previously only to be found scattered in the literature and now present a concise and easy tool to apply.

## Limitations and conclusion

Since the scavenging lesions in our cases were of no legal relevance and we collected the data retrospectively, they lack a systematic approach as suggested in our flowchart. However, they serve as an example of what the database from “everyday” casework looks like and emphasise the need to document lesions and detailed information about the circumstances and potential scavengers as comparative data for future work. Furthermore, the estimated PMIs are vague and the cause of death is unclear in several cases. Autopsies could not be performed in all of them.

Our study highlights the need for sophisticated documentation, particularly for distinction between human-inflicted injuries and scavenging, but also discrimination between cats and dogs, and between canines of different sizes. It also shows that the information typically collected at scenes of indoor scavenging is insufficient to perform a discrimination study. We therefore developed a decision diagram to help overcome these aspects and gather systematic information for future indoor death scenes with questionable scavenging activity.

## Key points

1. Indoor scavenging by cats and/or canines can complicate forensic investigations
2. We reviewed the scientific literature on indoor cat and dog scavenging
3. We present seven new cases of indoor scavenging
4. We propose a chart to identify, document and report indoor scavenging
5. A solid data basis will aid the distinction between cat and canine scavenging

## Data Availability

The data was generated or analysed within the framework of our study, based on unpublished case reports of forensic investigators. We present them anonymized.
